# Functional antibody responses to SARS-CoV-2 variants before and after booster vaccination among adults in Ghana

**DOI:** 10.3389/ebm.2025.10440

**Published:** 2025-07-21

**Authors:** F. D. Partey, A. N. A. Pobee, I. K. Damptey, F. Osei, M. M. A. K. Owusu-Amponsah, Y. A. A. Ansah, C. Ye, S. Bradfute, I. Hurwitz, P. K. Quashie, M. F. Ofori, A. K. Kusi, D. J. Perkins, G. A. Awandare

**Affiliations:** ^1^ Department of Immunology, Noguchi Memorial Institute for Medical Research, College of Health Sciences, University of Ghana, Accra, Ghana; ^2^ Center for Global Health, Department of Internal Medicine, University of New Mexico School of Medicine, University of New Mexico, Albuquerque, NM, United States; ^3^ West African Center for Cell Biology of Infectious Pathogens, College of Basic and Applied Sciences, University of Ghana, Accra, Ghana

**Keywords:** COVID-19 vaccination, COVID-19 booster, SARS-CoV-2, booster vaccination, COVID-19

## Abstract

COVID-19 booster vaccinations are needed to enhance waning immunity and the emergence of new variants. In Africa, where COVID-19 vaccine coverage is low, there is a paucity of data on COVID-19 vaccine-induced immunity, particularly in the post-omicron era. This study examined the functional activity of vaccine-induced antibody responses against different variants before and after booster vaccinations in adults in Ghana, between November 2022 and February 2023. SARS-CoV-2 nucleocapsid protein and spike receptor binding domain (RBD) antigen-specific IgG levels against different viral variants were determined in plasma. Plasma was tested for the ability to inhibit ACE-2 binding to RBD variants. N antigen-specific antibody levels were comparable between vaccinated and previously infected, but unvaccinated individuals. However, RBD IgG levels before booster vaccinations were significantly higher in vaccinated participants than in exposed, unvaccinated individuals, except for Omicron. RBD IgG levels remained unchanged after the booster in participants with three prior vaccine doses but were significantly higher than in those with only primary vaccinations (Wild type p = 0.0315, Alpha p = 0.0090, Beta p = 0.0020, Delta p = 0.0040) except Omicron (p = 0.09). Participants who received the Pfizer-BioNTech vaccine showed a significant increase (p < 0.05) in RBD IgG levels against all tested variants from baseline to 3 months. In contrast, participants who received the J&J vaccine only showed a significant increase in RBD IgG to Wildtype (p = 0.04), Alpha (p < 0.0001), and Beta (p < 0.0001), but not Delta and Omicron. The inhibition of ACE-2 binding and live virus neutralization titers were significantly higher in vaccinated individuals than in unvaccinated individuals before the booster (p < 0.001). Virus neutralization titers against Wildtype were significantly high 3 months after booster (p < 0.001), but neutralization titers against Omicron remained stable from baseline to 3 months after booster. Extended interval between vaccinations may enhance vaccine-induced antibody responses.

## Impact statement

Information on COVID-19 booster vaccine-induced immunity and the timing of booster vaccination for enhanced immunity against emerging viral variants in sub-Saharan Africa is scanty. This study provides knowledge on the efficacy of COVID-19 booster vaccines specifically within the African population by examining the functional activity of vaccine-induced antibody responses against different.

SARS-CoV-2 variants. This work offers new insights into COVID-19 vaccine effectiveness and durability in Ghanaian adults, showing that booster doses enhance antibody levels and cross-reactive responses, though they have limited impact on omicron-specific responses. It also finds that Pfizer-BioNTech 's mRNA vaccine is more effective than the J&J vaccine in boosting antibody responses. Our data supports extended dosing intervals for enhanced vaccine-induced responses. This finding has implications for guiding vaccination policies and resource allocation in resource-limited settings.

## Introduction

The global administration of COVID-19 vaccines significantly reduced mortality rates during the SARS-CoV-2 pandemic [1]. Vaccination further reduces the risk of severe disease [2–5], hospitalizations [6–8] and post-disease conditions [9, 10]. However, declining immune response following both natural infections and vaccinations, along with the emergence of viral variants with enhanced transmission, necessitates booster vaccinations to enhance and/or maintain immunity. In many advanced countries, additional booster vaccinations have been recommended. A meta-analysis examining the declining efficacy of COVID-19 vaccines by the number of doses against the SARS-CoV-2 Delta and Omicron variants found that protection is significantly enhanced and more durable after booster vaccinations compared to primary vaccination [11]. However, there remains a significant knowledge gap regarding the durability of vaccine-induced immune responses after booster vaccinations within the broader sub-Saharan African (SSA) population. Understanding the longevity of these immune responses within populations is crucial for informing vaccination strategies to mitigate future epidemics.

Several studies among Africans demonstrate that even among HIV uninfected individuals, there is chronic immune dysregulation when compared to other populations [12–15]. This observation is characterised by low CD4^+^ T cells count and a skewed CD4/CD8 ratio. Immune dysregulation is largely triggered by environmental factors [12, 16]. A key environmental factor is infection with helminths which attenuate responses to parasitic [17], bacterial [18, 19] and viral infections [20]. Similarly, helminth-driven immune dysregulation has been found to negatively affect vaccine efficacy in African populations [21, 22]. Helminth-infected individuals elicit reduced vaccine-induced immune responses against malaria [23], tetanus toxoid [24], and *Bacillus* Calmette-Guérin (BCG) [25]. The mechanisms by which co-infections attenuate vaccine-induced immune responses include systemic immunosuppression [26], depletion of immune cells [21] and increased activation of immune tolerogenic signals [22].

Earlier studies on both mRNA and adenoviral-vectored COVID-19 vaccines revealed high vaccine immunogenicity among African adult populations [27–30], comparable to studies from developed countries and other low- and middle-income countries (LMICs) with similar demographics. These studies demonstrated heightened antibody responses in previously infected individuals compared to those in infection-naïve individuals. However, these studies primarily reported on binding antibody titers and did not assess the functionality of vaccine-induced antibodies, such as virus-neutralizing antibody titers. Neutralization assays estimate the ability of circulating virus-specific antibodies to prevent viral entry into the host cells. Serum neutralizing antibody titers have been shown to strongly correlate with protection in both clinical and animal studies [31–33]. ACE-2 inhibition assays are surrogate neutralization assays that quantify the capacity of antibodies to block the binding of the SARS-CoV-2 spike protein to ACE-2 [34]. Together, these assays provide insights into the quality of vaccine-induced humoral responses and potential vaccine efficacy beyond antibody magnitude. Furthermore, the dynamics of immune responses to heterologous vaccination regimens after additional booster shots in SSA populations remain poorly understood.

Ghana was the first African country to receive nCoV-Chadox 1 (Oxford/AstraZeneca) vaccines from the COVID-19 Vaccines Global Access (COVAX) facility, a platform created to ensure equitable access to vaccines. mRNA-based (Pfizer-BioNTech BNT162b2 and Moderna mRNA-1273) and adenoviral-vectored [Sputnik V and Johnson & Johnson (J&J)] vaccines were introduced to the public as they became available. While the mRNA, AstraZeneca, and Sputnik V vaccines were administered in two doses, the J&J vaccine was primarily given as a single dose. Due to the logistical challenges in vaccine supply, the Ghana Health Service endorsed the use of different COVID-19 vaccines for the two-dose regimens, depending on the availability of vaccines in the country.

The population level COVID-19 vaccine efficacy within the African population outside clinical trials is unknown. Additionally, the impact of parasitic and other viral coinfections on vaccine-induced immune responses remains poorly defined.

In the present study, we examine the functional activity of COVID-19 vaccine-induced antibody responses against various SARS-CoV-2 variants after booster vaccinations among vaccinated and unvaccinated Ghanaian adults with prior SARS-CoV-2 infection.

## Materials and methods

### Study design and participant

The present study was conducted between November 2022 and February 2023. Previously vaccinated individuals ≥18 years who were willing to receive COVID-19 booster vaccines were enrolled in the study. Participants were recruited after informed consent, from vaccination centers around the University of Ghana, Legon, and its environs in the capital city, Accra. Blood samples were collected from participants before the last booster vaccination and 3 months after receiving the booster vaccination. Blood samples from unvaccinated individuals who were COVID-19-positive, either by PCR or rapid diagnostic test, were used as controls. Control samples were collected in April 2021 before the mass vaccination campaign was launched in Ghana. The samples were used as naturally exposed unvaccinated controls to examine vaccine-induced responses in naturally exposed individuals, as exposure was high in the study population. We randomly selected archived samples for which sufficient biological material was available for all assays. For all participants, blood was drawn into heparinized tubes and centrifuged at 800 × g for 5 min to separate plasma from the cellular fraction. Plasma was stored at −30°C until ready for use.

### SARS-CoV-2 antigens

The nucleocapsid (N) antigen used in the study was expressed in *Escherichia coli*, while the receptor binding domain (RBD) of the spike protein of the ancestral strain was expressed in freestyle 293F cell systems as described elsewhere [18, 35]. Alpha and Beta RBD were optimized for expression in the ExpreS2 platform as described [9, 36]. Delta RBD (Sino Biological # 40592-V08H115) and Omicron RBD (Sino Biological # 40592-V08H143) were expressed with a polyhistidine tag at the C-terminus in HEK293 cells.

### Enzyme-linked immunosorbent assay for SARS-CoV-2 antigens

An indirect ELISA was used to measure plasma antigen-specific IgG to N and RBD of the various SARS-CoV-2 variants as previously described [37, 38]. Nunc Maxisorp plates were individually coated with either 0.5 μg/mL of N antigen, Ancestral RBD, Alpha RBD, and Beta RBD or 1 μg/mL of Delta RBD and Omicron RBD at 4°C overnight. Plates were then washed with 0.05% Tween in PBS and blocked with 1% BSA in PBS for an hour at room temperature (RT) before use. Subsequently, plasma was added to the wells in duplicate at a dilution of 1:100 for all antigens and incubated for 1 h at RT. Plates were washed and HRP-conjugated rabbit anti-human secondary antibody (1:3000) was added and incubated at RT for 1 h. Wells were developed by adding 3,3′,5,5′-tetramethylbenzidine (TMB) as substrate and stopped with 2 N H_2_SO_4_. Absorbance in each well was read at 450 nm using a microplate reader.

To establish a cutoff for seropositivity against any antigen, pre-pandemic samples were included in the assay as negative controls, and the COVID-19 convalescent plasma pool was used as a positive control. Positive and negative controls were included on each plate to account for plate-to-plate and inter-assay variations.

### SARS-CoV-2 inhibitor screening

Plasma from a subset of participants (vaccinated n = 21, unvaccinated n = 55) was screened for its ability to inhibit ACE-2 binding to RBD variants using the SARS-CoV-2 Variant Inhibitor Screening Kit (R&D Systems, #VANC00B) following the manufacturer’s protocol. Briefly, 96 well plates were coated with His-Tag capture antibody and incubated at 4°C overnight. The plates were washed and blocked for 1 h at 37°C. RBD variants (wild type, alpha, delta, and omicron) were added to different wells in plates and incubated for 1 h at RT. After incubation, the plates were washed, plasma was added to the wells at a dilution of 1:100 and then incubated for 1 h at RT. Biotinylated human ACE-2 was added to wells followed by 90 min incubations. Following washing, streptavidin-HRP conjugate was added to plates and incubated for 30 min at RT. The plates were washed, substrate solution added, followed by incubation at RT for 20 min. The reaction was stopped with 2 N H_2_SO_4_ and the absorbance was read at 450 nm.

### Plaque reduction neutralization test

The same plasma from vaccinated individuals (n = 55) tested in the ACE inhibition above was also tested in a live virus neutralization assay against the wild-type and Omicron viral variants (BEI Resources; National Institute of Allergy and Infectious Diseases) as described previously [39]. In summary, the isolates were diluted to 50–100 plaque forming units/200 μL in minimum essential medium supplemented with 2.5% heat inactivated fetal calf serum (viral growth medium). Test plasma was heat inactivated at 56°C for 30 min and 2-fold serial dilutions prepared starting from a dilution of 1:40. The diluted plasma was added to equal volumes of diluted virus and incubated for 1 h. The plasma-virus culture was next added to Vero E6 cells and incubated at 37°C for 2 h. Virus-only mixtures were used as controls. Following virus aspiration, cells were overlaid with virus overlay medium by mixing equal volumes of 2x antibiotic-containing virus growth medium and 2% agarose gel and incubated at 37°C for 48 h. Cells were fixed with 4%formaldehyde overnight at 4°C. subsequently, the overlay was removed, and cells were stained with 0.5% crystal violet for 2 min, washed, and dried. Plaques were enumerated to estimate 80% plaque reduction neutralization titers (PRNT80). We determined PRNT80 as a more stringent reduction threshold to detect robust protective neutralization titers and reduce the risk of background cross-reactive antibodies

### Statistical analysis

Statistical analyses were performed using GraphPad Prism software version 10.0. The Kruskal–Wallis test was used to compare IgG levels between the groups. Two-way ANOVA was used to compare IgG levels before and after vaccination between and within different participant groups. All comparisons were two-tailed and P values < 0.05 were regarded as statistically significant.

## Results

### Patient characteristics

This study recruited 86 vaccinated individuals between November 2022 and February 2023. The vaccinated participants comprised 31 females and 55 males with an age range of 18–60 years (Table 1). Most volunteers were aged–18–25 (55.82%). At enrolment, the vaccinated participants had previously received at least one shot of one vaccine type, including AstraZeneca (46.51%), J&J (22.1%), Moderna (17.44%), or Pfizer-BioNTech (10.5%). The mean number of days from the primary vaccination to baseline sampling was 461, ranging from 45 to 734 days. The mean number of days from the last vaccination to baseline sampling was 372, ranging from 45 to 666 days, and was significantly higher among participants who had received two vaccine doses than among those who had received only one dose in the study (Supplementary Figure S1). The unvaccinated controls were previously infected individuals with a confirmed RT-PCR or antigen-RDT diagnosis, who were enrolled in a related study in March 2021.

**TABLE 1:** Characteristics of vaccinated and unvaccinated participants.

Variables	Vaccinated	Unvaccinated	P - value
n	86	36	
Age, yr, *n* (%)			
18–25	48 (55.82)	12 (33.33)	p = 0.004
26–35	22 (25.58)	7 (19.44)	
36–45	8 (9.3)	9 (25)	
46–60	8 (9.3)	8 (22.22)	
Sex, *n* (%)			
Female	31 (36.05)	22 (64.71)	p = 0.003
Male	55 (63.95)	12 (35.29)	
Previous vaccination, *n* (%)			
AstraZeneca	40 (46.51)	n. a	
J&J	19 (22.10)		
Moderna	15 (17.44)		
Pfizer	9 (10.47)		
Others	3 (3.49)		
Number of previous vaccine doses *n* (%)			
1	19 (20.10)	n. a	
2	52 (60.47)		
3	15 (17.44)		
Time since first vaccine dose, days mean (range)	461 (45–734)	n. a	
Time since first vaccine dose, days mean (range)	372 (45–666)		

Abbreviation: n.a, not applicable.

### SARS-CoV-2 RBD-specific antibodies are boosted with vaccination in naturally exposed individuals

**FIGURE 1:**
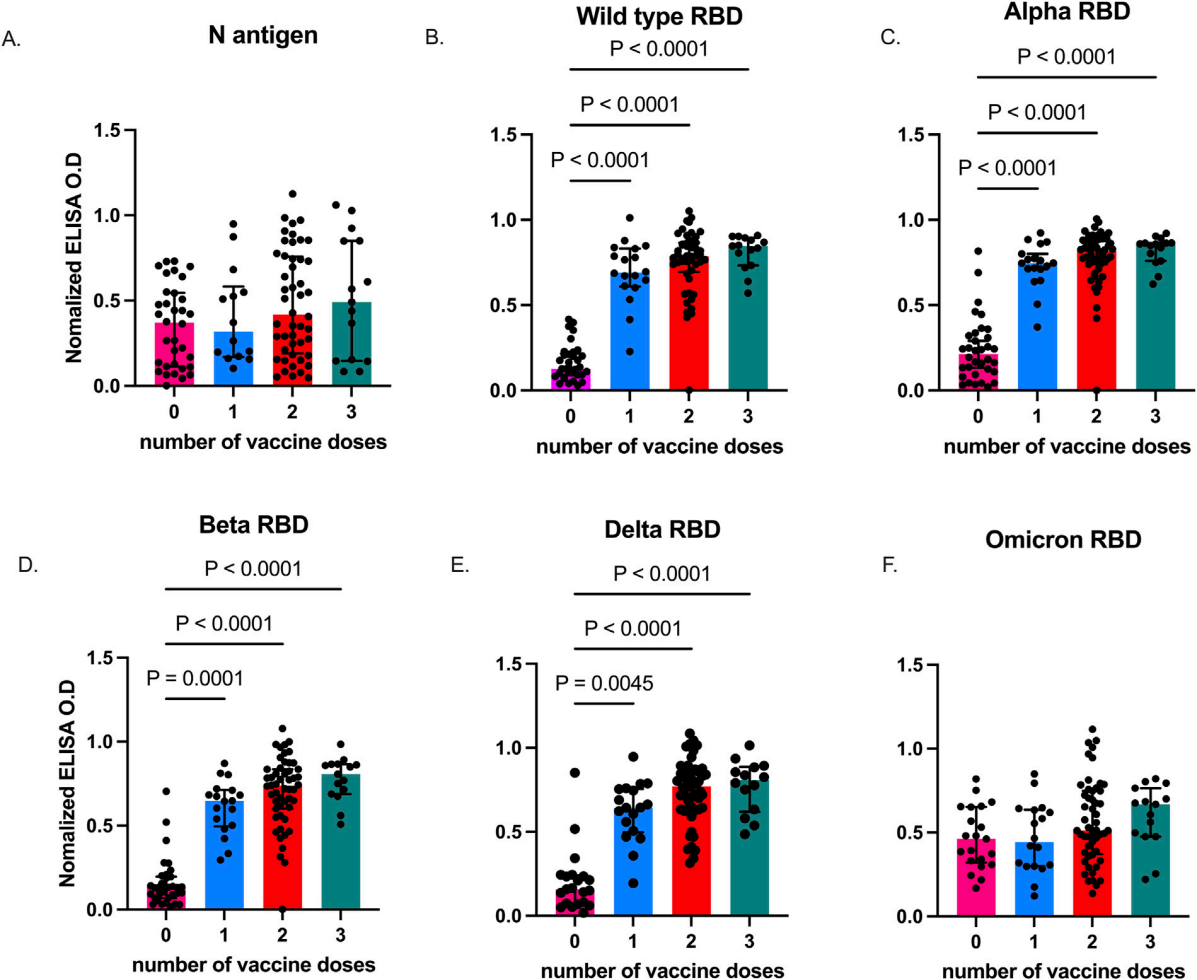
SARS-CoV-2-specific IgG levels in vaccinated and unvaccinated adults. Data plots showing plasma N antigen levels **(A)**, and plasma RBD IgG levels against Wild type **(B)**, Alpha **(C)**, Beta **(D)**, Delta **(E)**, and Omicron **(F)**. Bars indicate the median and error bars represent the interquartile range. P values <0.05 are stated on the graphs. Unvaccinated controls were designated as those who received zero vaccine doses.

Previous seroepidemiological studies in Accra and its surrounding areas found high SARS-CoV-2 exposure among the general population [40]. Thus, we first measured antibodies against the nucleoprotein (N) in plasma from all study participants at baseline, before they received booster vaccination and plasma from individuals with confirmed SARS-CoV-2 collected in 2021 were also included as unvaccinated controls. Plasma N antigen-specific IgG levels were comparable between vaccinated individuals who had received at least a single dose of COVID-19 vaccine and exposed unvaccinated individuals (p = 0.2095 (Figure 1A). In contrast, RBD antibodies against the different viral strains (Figures 1B–E) were significantly higher (p < 0.0001) in the vaccinated participants at enrolment when compared to exposed, unvaccinated individuals, except for Omicron (p = 0.1161, Figure 1F).

**FIGURE 2:**
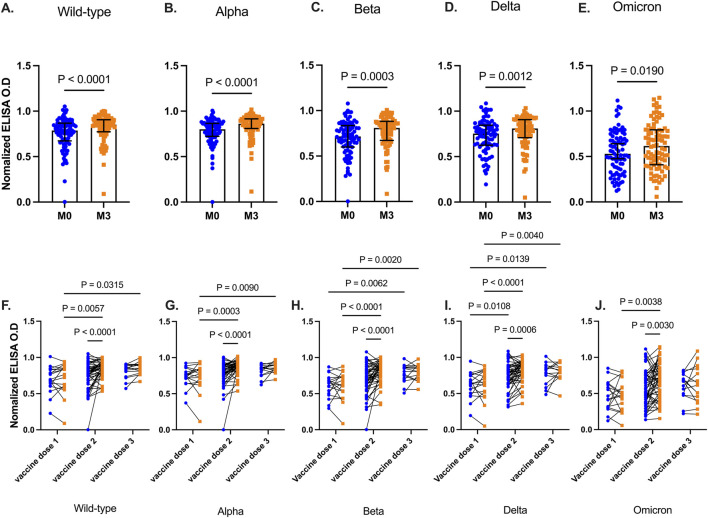
SARS-CoV-2 RBD levels before and 3 months after booster vaccinations. Data plots showing plasma RBD IgG levels against Wild type **(A)**, Alpha **(B)**, Beta **(C)**, Delta **(D)**, and Omicron **(E)**. Bars indicate the median and error bars represent the interquartile range. Line plots showing RBD IgG levels before and 3 months post-booster vaccination against Wild type strain **(F)**, Alpha **(G)**, Beta **(H)**, Delta **(I)**, and Omicron **(J)** variants. Participants are grouped based on the number of COVID-19 vaccines they had received before receiving the additional booster vaccination. Blue-filled circles and orange-filled squares represent IgG levels at baseline and month 3 respectively. Comparisons were done before and after across the number and vaccines received. P values <0.05 are stated on the graphs are stated on the graphs.

We determined levels of vaccine-induced antibody responses, 3 months post-booster administration. Plasma collected before, and after, booster vaccinations were tested for antibodies against RBD antigens from the five SARS-CoV-2 variants. Plasma RBD-IgG levels increased significantly 3 months after the booster vaccination for Wild type (p < 0.0001), Alpha (p < 0.0001), Beta (p = 0.0003), Delta (p = 0.0012), and Omicron (p = 0.0190) (Figures 2A–E).

More granularity was achieved when participants were then grouped based on the number of COVID-19 vaccine doses they had received prior to the additional booster (Figures 2F–J). There was no significant difference between RBD IgG levels at baseline and 3 months after the booster, for all tested viral strain antigens (Wild type p = 0.64, Alpha p = 0.95, Beta p = 0.99, Delta p = 0.91, Omicron p = 0.89) in participants who received only a single dose of the COVID-19 vaccine. In contrast, individuals who had received two vaccine doses and the booster showed a marked increase (Wild type, Alpha, Beta, p < 0.001, Delta p = 0.0006, Omicron p = 0.003) in RBD IgG levels against all viral strain antigens. Similar results were observed 3 months post booster in participants with three prior vaccine doses except for Omicron (p = 0.80), RBD IgG levels increased modestly after the booster. However, for the other viral variants, RBD IgG levels were significantly higher than in those with only primary vaccinations (Wild type p = 0.032, Alpha p = 0.009, Beta p = 0.002, Delta p = 0.004)

### BNT162b2 Pfizer/Bio-n-tech booster vaccination enhances durability of cross-strain reactive antibodies 3 months post booster vaccination

**FIGURE 3:**
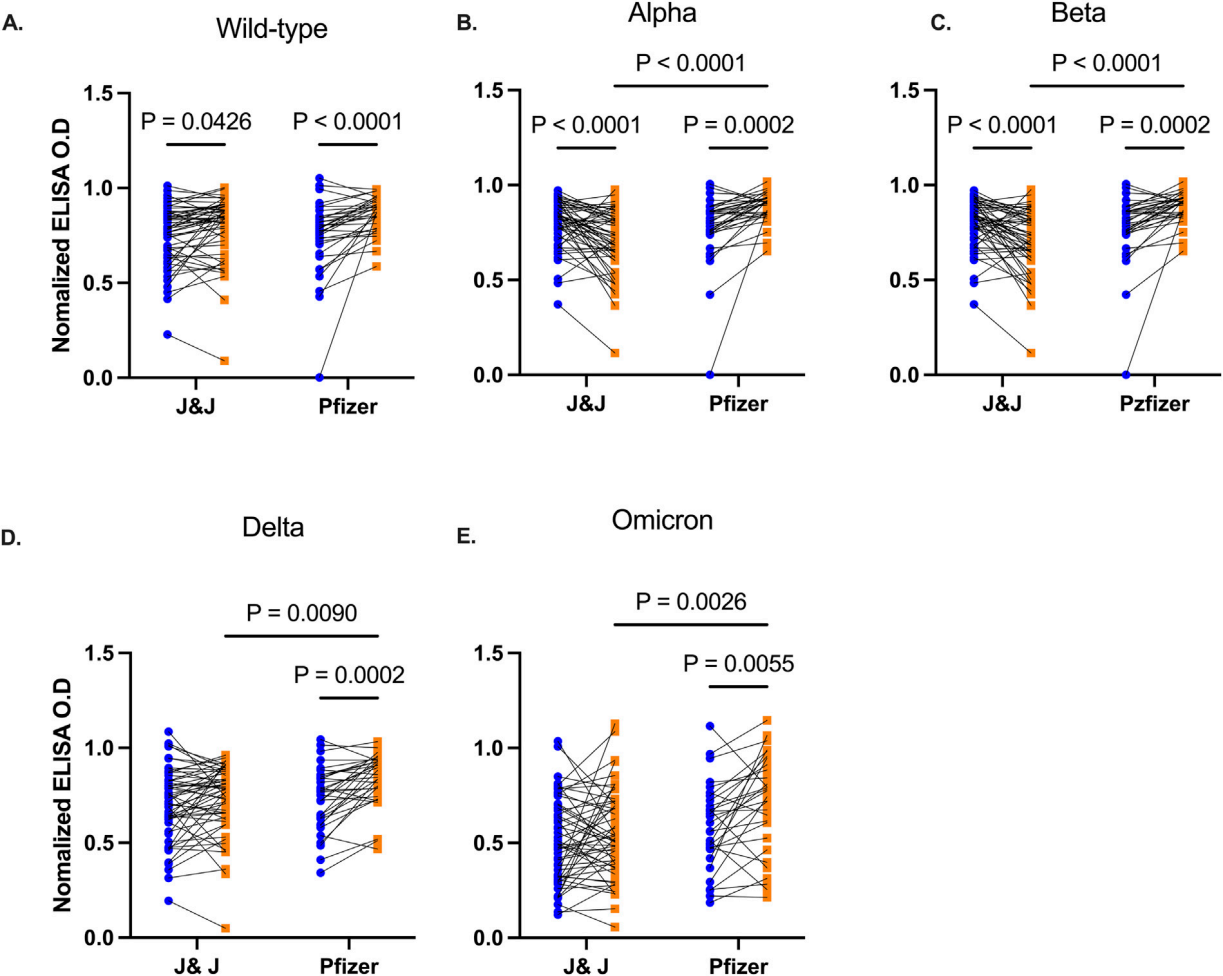
Comparative RBD IgG levels between participants receiving Pfizer-BioNTech and J&J vaccine. Line plots showing RBD IgG levels before and 3 months post booster vaccination against Wild type **(A)**, Alpha **(B)**, Beta **(C)**, Delta **(D)**, and Omicron **(E)** variants. Participants are grouped based on the type of booster vaccine they received. Blue-filled circles and orange-filled squares represent IgG levels at baseline and month 3 respectively. P values <0.05 are stated on the graphs.

We then examined the durability of vaccine-induced antibodies based on the type of booster, Pfizer-BioNTech or J&J, the participants received. Anti-RBD IgG antibody levels were comparable between individuals prior to receiving the booster. Three months following booster, participants (n = 34) who received the Pfizer-BioNTech booster elicited a marked increase in their antibody levels against RBD of all the test viral variants (Wild type p < 0.0001, Alpha p = 0.0002, Beta p = 0.0002, Delta p = 0.0002, Omicron p = 0.0055, Figures 3A–E). Recipients (n = 55) of the J&J booster also showed an increase in anti-RBD antibody levels against the Wild type (p = 0.043), Alpha (p < 0.0001) and Beta variants (p < 0.0001) but IgG levels against Delta (p = 0.12) and Omicron (p = 0.22) variants remained unchanged relative to pre-booster levels.

### Booster vaccination enhances cross-strain viral-neutralizing antibodies

**FIGURE 4:**
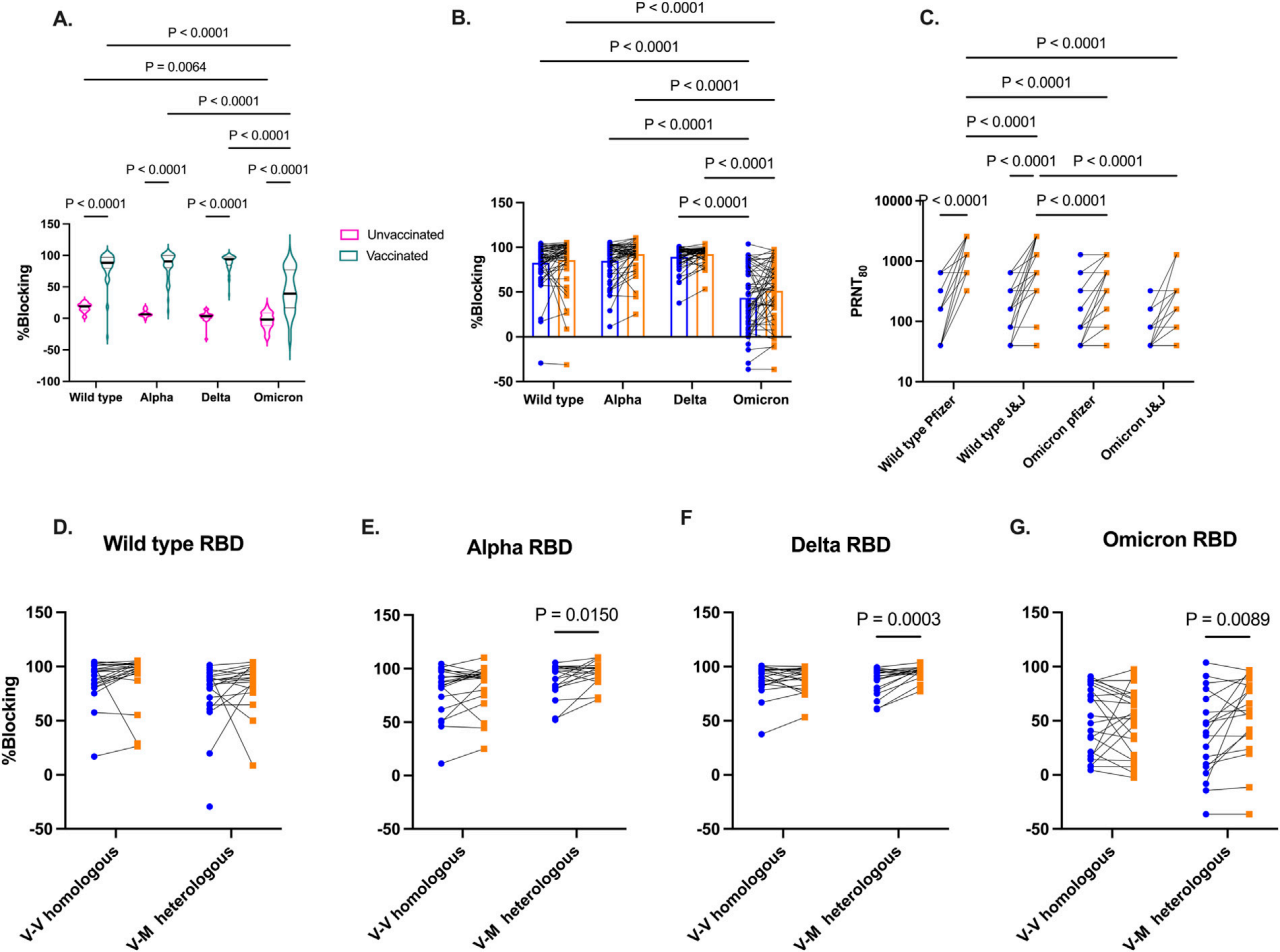
Competitive inhibition of ACE-2 binding to RBD and live virus neutralization. *In vitro* inhibition of ACE-2 binding to RBD by plasma from vaccinated and unvaccinated volunteers **(A)**. Percentage blocking among vaccinated individuals before and 3 months after booster vaccinations **(B)**. Live virus neutralization by plasma from vaccinated individuals against wild-type and Omicron variants based booster type received (Pfizer or J&J) **(C)**. Percentage blocking of ACE-2 binding among vaccinated individuals grouped by homologous and heterologous booster vaccination regimen against RBD from Wild type **(D)**, Alpha **(E)**, Delta **(F)**, and Omicron **(G)**. Blue-filled circles and orange-filled squares represent IgG levels at baseline and month 3 respectively. P values <0.05 are stated on the graphs.

The functionality of antibodies that would block the binding of SARS-CoV-2 RBD to the angiotensin-converting enzyme (ACE-2) receptor on host cells was assessed by screening plasma from two distinct groups of participants – those who were unvaccinated but had been exposed to SARS-CoV-2, and those who had been vaccinated. Plasma collected from these participants was screened for inhibition of ACE-2 binding to RBD from Wild type, Alpha, Delta, and Omicron utilizing a commercially available kit. As expected, vaccinated individuals before booster show higher inhibition of ACE-2 binding to Spike RBD across the four SARS-CoV-2 strains compared to those who were exposed but unvaccinated (Figure 4A). In vaccinated group, binding inhibition was found to be lowest against Omicron Spike RBD.

The level of SARS-CoV-2 Spike RBD binding antibodies was examined again 3-month later following booster administration (Figure 4B). While there was an increase in Spike RBD binding antibodies levels across all four viral variants post-booster, the levels of inhibitory antibodies were not found to be significant (Wild type p = 0.47, Alpha p = 0.08, Delta p = 0.48, Omicron (p = 0.08). Furthermore, pre- and post-booster plasma were tested in a plaque reduction neutralization test (PRNT) to demonstrate *in vitro* neutralization against wildtype and Omicron strains (Figure 4C) Boostered plasma showed a significant enhancement (p < 0.0001) in neutralizing wildtype SARS-CoV-2 among recipients of both the Pfizer and Janssen booster shots. However, 3 months post-booster, the neutralizing IgG levels were notably higher in Pfizer recipients compared to those who received the Janssen booster (p < 0.0001). In contrast, Omicron neutralizing IgG levels did not significantly increase in both recipient groups from baseline to 3 months post booster. Wildtype neutralizing IgG levels were markedly high than Omicron neutralizing IgG levels 3 months post booster (p < 0.0001).

Since most of our participants received viral-vectored vaccines as primary vaccinations, we grouped participants into homologous and heterologous groups. Homologous vaccination received viral-vectored vaccines as both primary and booster vaccination. Heterologous vaccination received viral-vectored and were subsequently boostered with the mRNA-based Pfizer vaccine. ACE-2 inhibitory antibody levels against the Spike RBD of the viral variants was examined 3 months post booster (Figures 4D–G). No difference was seen in the level of inhibitory IgG levels against the 4 SARS-CoV-2 variants for the homologous group. However, heterologous boosting increased inhibitory antibody levels Against Alpha (p = 0.02), Delta (p = 0.0003), and Omicron (p = 0.0089).

## Discussion

The emergence of SARS-CoV-2 variants and waning immunity after infection or vaccination necessitated vaccine boostering to maintain durable immune responses [41]. In sub-Saharan Africa (SSA), there is a paucity of data on the real-world efficacy and durability of COVID-19 vaccine responses, particularly in the post-Omicron era. In the present study, we examined the magnitude and functionality of SARS-CoV-2-specific antibody responses among vaccinated Ghanaian adults 3 months after receiving either Pfizer-BioNTech or J&J booster immunizations and compared these against responses in unvaccinated individuals with natural SARS-CoV-2 infections.

Before the booster, anti-N antibodies were comparable between vaccinated and unvaccinated individuals with a known history of infection. Consistent with findings from different geographical settings [42, 43], vaccination did not impact anti-N antibodies among our participants. This suggests that anti-N antibodies were acquired from natural infections rather than from vaccination. We note a high proportion of vaccinated individuals were seropositive for N antibodies, reinforcing earlier findings that undetected asymptomatic SARS-CoV-2 infection rates in Ghana [37, 40] and other countries in SSA [44] were high.

At baseline, vaccinated individuals had significantly high levels of anti-RBD IgG levels against Ancestral, Alpha, and Delta (p < 0.05) but not Omicron (p = 0.12), when compared to unvaccinated controls, suggesting the presence of vaccination-induced cross-reactive antibodies. Baseline anti-RBD IgG levels against Omicron did not differ either by the number of prior vaccine doses received or between previously vaccinated and unvaccinated individuals sampled in 2021. Omicron was first reported in Botswana and South Africa in late 2021 [45], and was already in circulation in Ghana at the time of enrolment [46]. The Omicron variant is highly mutated compared to the Delta and other earlier variants. Mutations occur in both structural and non-structural proteins [47] which increases its transmissibility and enhances immune evasion from previous immunity [48–50]. Due to the mutations in the Omicron RBD, it is not surprising we observed reduced omicron-specific IgG binding levels in the vaccinated and unvaccinated groups. Our findings are similar to previous studies in different populations showing reducing binding and neutralizing antibody against Omicron [51, 52].

Overall, increased anti-RBD IgG levels against all SARS-CoV-2 strains in individuals who had previously received two or three vaccine doses, compared to those who had received only a single dose. Despite the vaccinated individuals’ seeming exposure to SARS-CoV-2, one booster dose did not significantly boost RBD IgG levels beyond baseline levels. Since participants were sampled 3 months after receiving their booster, it is possible that antibody levels had peaked earlier and declined more rapidly in participants who received one vaccine dose compared to those who had received at least two vaccine doses. A previous study in Ugandan adults who received the AstraZeneca vaccine [30] reported increase in RBD-specific IgG and IgA levels 14 days after the second vaccine dose, peaking at day 28, followed by a gradual decline in IgG levels.

The administration of a fourth booster dose to participants who were vaccinated three times did not significantly increase RBD IgG levels against all the tested viral strains. This raises a question regarding the timing of booster vaccination. The median time since the last vaccination before administering the booster in the present study was significantly higher among individuals who had received two vaccine doses relative to those who had just received a single dose (Supplementary Figure S1). It is well established that an extended dosing duration between vaccinations could enhance vaccine immunogenicity [53–55]. Another explanation for this observation is that within the settings of the study where natural exposure is high, few vaccine boosters may be required to reach the threshold of antigen stimulated immunity. Under such circumstances, natural infections may offer additional booster effects. Similar observations have been made in other populations within sub-Saharan Africa [27]. In resource-limited settings like SSA with high SARS- CoV-2 exposure, our data suggests that fewer booster vaccinations at longer intervals may help maintain robust antibody responses. However, it is critical to consider the specific needs of target populations, such as the elderly and individuals with comorbidities, who are at the highest risk of severe disease.

Functional antibody responses were assessed using either their capacity to inhibit the binding of a soluble form of the ACE-2 to the RBD from the Wild type, Alpha, Beta, Delta, and Omicron viral variants, or their capacity to neutralize viruses expressing the spike proteins from the Wild type and Omicron SARS-CoV-2 variants. Inhibition to ACE-2 binding to RBD from all tested variants markedly increased among the vaccinated individuals relative to the unvaccinated individuals. However, inhibition of ACE-2 binding to the Omicron RBD was significantly lower compared to the other variants tested. Similarly, cross-strain viral neutralizing activity among the vaccinated individuals was enhanced with booster vaccinations. Our findings are in line with prior studies showing reduced neutralization against Omicron than earlier variants [55].

Participants who received heterogeneous vaccine doses (J&J followed by Pfizer-BioNTech booster) displayed significantly higher (p < 0.05) antibody responses quantitatively and qualitatively compared to those who received homologous vaccine doses (J&J followed by J&J booster). The findings of the present study are consistent with earlier research which demonstrated that [56] mRNA vaccines, when used in heterologous booster regimens, elicit stronger IgG binding and neutralizing activities against Wild-type, Delta and Omicron variants compared to viral-vectored vaccines.

There are several limitations to our study. The limited age range of our study participants prevented us from examining the age effects on vaccine-induced antibody responses. A second limitation is 3-month interval between the booster vaccination and the blood draw. This prevented us from tracking antibody levels during the intervening period. Nonetheless, we still noted the significant increase in IgG levels in recipients of the Pfizer-BioNTech booster in comparison to those receiving the J&J booster. Future experiments would involve longitudinal studies to track IgG antibody levels and neutralization activities at multiple time points post-booster, and to compare these activities in mRNA and viral-vectored vaccines. A third limitation is the variation in the enrolment timelines between our vaccinated participants and unvaccinated controls. When the vaccinated participants were recruited in 2022, Ghana had already experienced four waves of COVID-19 driven by the emergence of new SARS- CoV-2 variants. The consistent levels of anti-N IgG levels in vaccinated individuals before and after booster vaccination would suggest that these participants were not exposed to SARS-CoV-2 in the intervening period. However, we cannot rule out the possibility that vaccinated individuals may have been infected since 2021, when the unvaccinated cohort was recruited. In addition, we noted a significant difference in sex between vaccinated and unvaccinated cohorts. However, we do not observe sex-dependent difference in variant-specific RBD IgG levels from baseline to 3 months post booster (Supplementary Figure S2).

In conclusion, we have shown that booster vaccination increases cross-strain viral IgG binding and neutralizing activity compared to natural infections in Ghanaian adults. Extended interval between vaccinations enhances vaccine-induced antibody responses, and booster vaccinations with the mRNA Pfizer-BioNTech vaccine were more effective than the vectored J&J vaccine.

## Data Availability

The original contributions presented in the study are included in the article/Supplementary Material, further inquiries can be directed to the corresponding author.
