# Supplementary material for: Functional antibody responses to SARS-CoV-2 variants before and after booster vaccination among adults in Ghana
Source: Exp Biol Med (Maywood). 2025 Jul 21;250:10440. doi: 10.3389/ebm.2025.10440 (PMC12318880; doi:10.3389/ebm.2025.10440)
Supplement: Supplementary file 1 [file Presentation1.pdf]

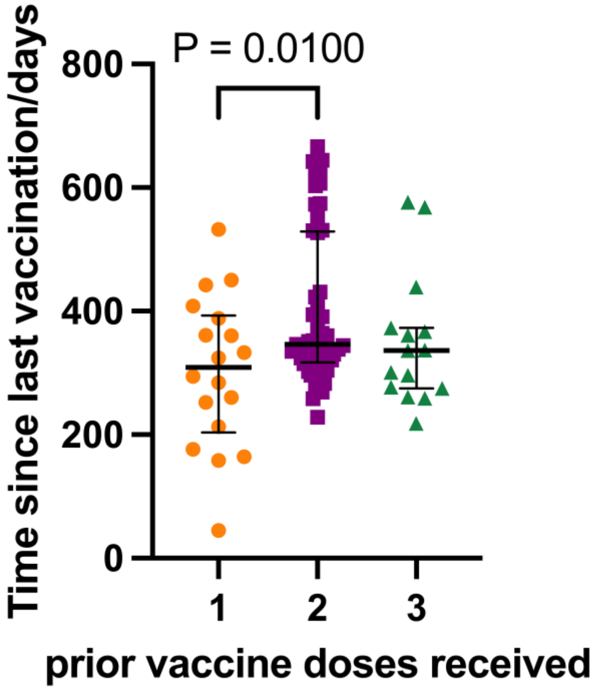


**Supplementary Figure 1**: Duration since last vaccination to baseline compared between recipients of one (orange-filled circles), two (purple-filled squares) and three (green-filled triangles) vaccine doses. Bars indicate the median and error bars represent the interquartile range. P values < 0.05 are stated on the graphs.

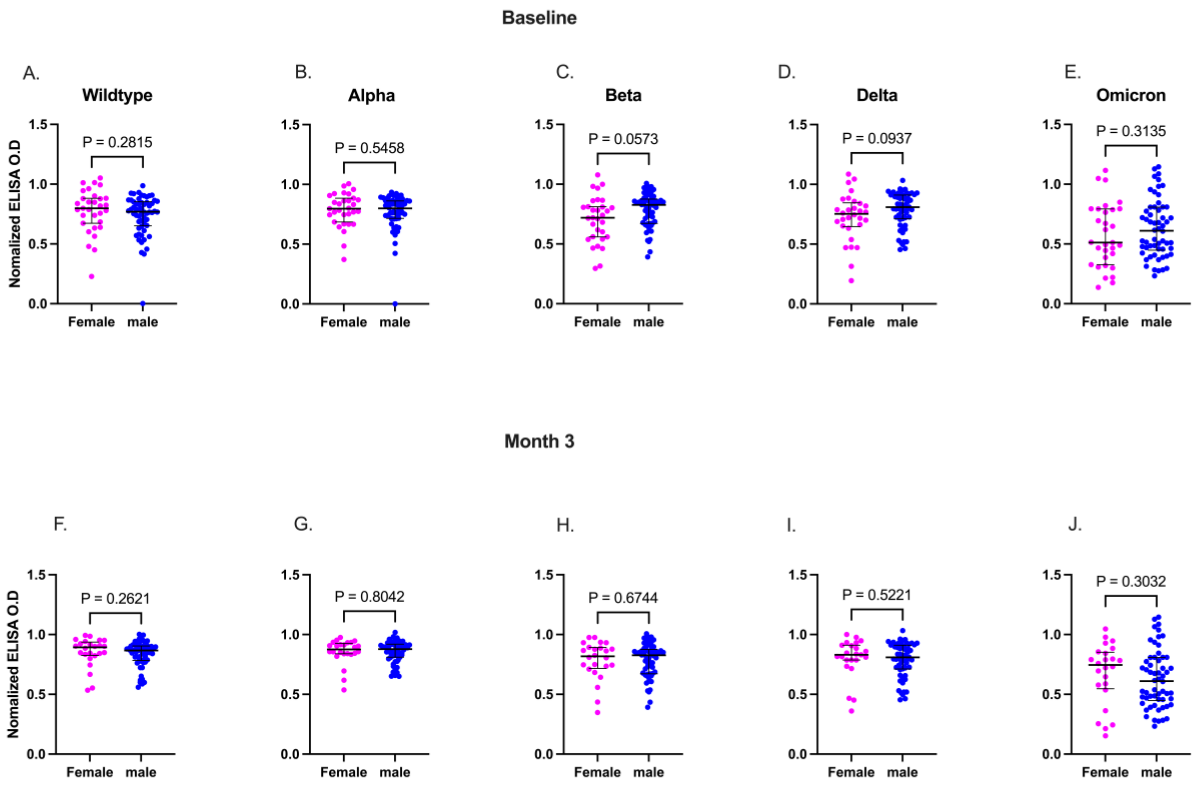


**Supplementary Figure 2:** Analysis of variant-specific RBD IgG levels between females and males at baseline(A-D) and 3 months post booster vaccination (F-J). Females are represented in pink-filled circles and males in blue-filled circles. Bars indicate the median and error bars represent the interquartile range. P values are stated on the graphs.
